# A method for genotyping elite breeding stocks of leaf chicory (*Cichorium intybus* L.) by assaying mapped microsatellite marker loci

**DOI:** 10.1186/s13104-015-1819-z

**Published:** 2015-12-30

**Authors:** Andrea Ghedina, Giulio Galla, Thierry Cadalen, Jean-Louis Hilbert, Silvano Tiozzo Caenazzo, Gianni Barcaccia

**Affiliations:** DAFNAE, Laboratory of Plant Genetics and Breeding, University of Padua, Campus of Agripolis, 35020 Legnaro, PD Italy; Agro-food and Biotechnology Laboratory, Charles Viollette Research Institute, Université Lille1 Sciences et Technologies, GIS CARTOCHIC, Cité Scientifique, 59655 Villeneuve d’Ascq, France; T&T Produce, S.S. Romea 77/bc, 30015 Sant’Anna Di Chioggia, VE Italy

**Keywords:** Radicchio, MAB, Inbred lines, SSR markers, Genotyping

## Abstract

**Background:**

Leaf chicory (*Cichorium intybus* subsp. *intybus* var. *foliosum* L.) is a diploid plant species (2*n* = 18) of the Asteraceae family. The term “chicory” specifies at least two types of cultivated plants: a leafy vegetable, which is highly differentiated with respect to several cultural types, and a root crop, whose current industrial utilization primarily addresses the extraction of inulin or the production of a coffee substitute. The populations grown are generally represented by local varieties (*i*.*e*., landraces) with high variation and adaptation to the natural and anthropological environment where they originated, and have been yearly selected and multiplied by farmers. Currently, molecular genetics and biotechnology are widely utilized in marker-assisted breeding programs in this species. In particular, molecular markers are becoming essential tools for developing parental lines with traits of interest and for assessing the specific combining ability of these lines to breed F1 hybrids.

**Results:**

The present research deals with the implementation of an efficient method for genotyping elite breeding stocks developed from old landraces of leaf chicory, Radicchio of Chioggia, which are locally dominant in the Veneto region, using 27 microsatellite (SSR) marker loci scattered throughout the linkage groups. Information on the genetic diversity across molecular markers and plant accessions was successfully assessed along with descriptive statistics over all marker loci and inbred lines. Our overall data support an efficient method for assessing a multi-locus genotype of plant individuals and lineages that is useful for the selection of new varieties and the certification of local products derived from Radicchio of Chioggia.

**Conclusions:**

This method proved to be useful for assessing the observed degree of homozygosity of the inbred lines as a measure of their genetic stability; plus it allowed an estimate of the specific combining ability (SCA) between maternal and paternal inbred lines on the basis of their genetic diversity and the predicted degree of heterozygosity of their F1 hybrids. This information could be exploited for planning crosses and predicting plant vigor traits (*i*.*e*., heterosis) of experimental F1 hybrids on the basis of the genetic distance and allelic divergence between parental inbred lines. Knowing the parental genotypes would allow us not only to protect newly registered varieties but also to assess the genetic purity and identity of the seed stocks of commercial F1 hybrids, and to certificate the origin of their food derivatives.

**Electronic supplementary material:**

The online version of this article (doi:10.1186/s13104-015-1819-z) contains supplementary material, which is available to authorized users.

## Background

Cichorium (*Cichorium intybus* subsp. *intybus* var. *foliosum* L.) comprises diploid plant species (2*n* = 18) belonging to the Asteraceae family, subfamily Cichoriodeae, tribe Lactuceae or Cichorieae. These species are biennial or, in the wild, perennial species [1]. They are naturally allogamous due to an efficient sporophytic self-incompatibility system. In addition, outcrossing is promoted by a floral morpho-phenology unfavorable to selfing in the absence of pollen donors (*i*.*e*., proterandry, wherein the anthers mature before the pistils) and a favorable competition of allo-pollen grains and tubes (*i*.*e.*, pollen that is genetically diverse from that produced by the seed parent, usually called auto-pollen) [2]. Long appreciated as medical plants by the ancient Greeks and Romans, *Cichorium* spp. are currently among the most important cultivated vegetable crops. They are generally used as components in fresh salads or, more rarely, cooked according to local traditions and alimentary habits [1].

Lacking comprehensive, homogeneous, sufficiently detailed, and univocal data on horticultural production and trade, it is difficult to give reliable figures on the diffusion and economic importance of this culture in Europe, where it is predominantly grown [1]. In recent statistics on the European market [3], chicory is often included under the general heading “salads” or considered together with lettuce, which is by far the most important leafy vegetable on both a European and world-wide scale. On the basis of accessible data [3], however, it is possible to determine that chicory is produced almost exclusively by Belgium, France, Italy, and the Netherlands [3]. Although chicory does not contribute greatly to each country’s total agricultural income, in the north eastern regions of Italy, it accounts for 87 % of the national acreage and 84 % of the national production of the red or variegated chicory known as “Radicchio”, which traditionally includes all the cultivar groups with leaf commercial products. This particular type of chicory is now receiving greater attention in Europe and the USA, where its cultivation originated several years ago, and is becoming increasingly subjected to evaluation because its red or variegated leaves are appreciated as a component of ready-to-eat salads [1].

The materials grown are generally represented by actively cultivated local populations (*i*.*e*., landraces) with high variation and adaptation to the natural and anthropological environment where they originated, and have been yearly selected and multiplied by farmers [1]. These populations are maintained by farmers through phenotypical selection based on their own criteria and occasionally on the exploitation of controlled hybridizations of different types to obtain recombinant genotypes that exhibit superior agronomic and commercial traits [4]. However, conventional plant breeding methods for hybridizing and selecting plants on the basis of observed phenotypes are not the only methods used by plant breeders. Currently, molecular genetics and biotechnology are widely utilized in breeding programs of the vast majority of crop plant species. Indeed, molecular markers are nowadays essential tools to select pure or inbred lines with qualitative traits of agricultural interest by marker-assisted selection (MAS) and also to predict the specific combining ability of parental lines to breed F1 hybrid varieties in marker-assisted breeding (MAB) schemes. In leaf chicory, molecular markers can find utility for assessing the degree of homozygosity of parental inbred lines, as a measure of their genetic stability, and also for predicting the degree of heterozygosity of their F1 hybrids, as an estimate of the specific combining ability on the basis of the genetic diversity between maternal and paternal inbred lines.

Here we describe a method for genotyping elite breeding stocks—inbred lines—using microsatellites, or SSR (simple sequence repeat) markers. Among the different PCR-derived molecular systems, SSR markers are suitable for population genetics studies and marker-assisted selection programs because they have several desirable features, such as a high level of reproducibility, co-dominant heredity, and no need for high throughput technology [5]. Moreover, they provide loci for simple and accurate individual typing in any species and display a high level of polymorphism and widespread distribution in the genomes [6]. Differently from other single-locus marker systems, like for instance SNP (single nucleotide polymorphisms) markers, microsatellite-based markers require a much less preliminary genomic information and bioinformatic characterization for their exploitation in a given species [7].

A large-scale application of molecular marker techniques, including amplified fragment length polymorphism (AFLP) and random amplified polymorphic DNA (RAPD), were used to construct the first genetic linkage maps of *C. intybus* [8, 9]. In 2010, a new genetic linkage map for *C. intybus* was constructed by using SSR markers [10]. This consensus genetic map, which includes nine homologous linkage groups (LGs), was obtained after the integration and ordination of the molecular marker data deriving from one witloof chicory and two industrial chicory progenies.

The aim of our study was to develop a method for the genetic characterization of elite inbred lines of the “Red of Chioggia” chicory using mapped SSR markers with a particular emphasis on the assessment of the genetic stability within (*i.e.,* observed degree of homozygosity) and genetic diversity between paternal and maternal lines (*i*.*e*, expected degree of heterozygosity of their F1 hybrids). Information derived from the application of this method should then be exploited for planning crosses and predicting plant vigor traits (*i*.*e*., heterosis) of experimental F1 hybrids of leaf chicory on the basis of the genetic distance and allelic divergence between parental inbred lines. Knowing the parental genotypes would allow us not only to protect newly registered varieties but also to assess the genetic purity and identity of the seed stocks of commercial F1 hybrids, and to certificate the origin of their food derivatives.

Basic genetic variation and differentiation statistics computed for single locus, across LGs and plant accessions are presented and discussed. Overall data support an efficient method for assessing a multi-locus genotype of plant individuals and lineages, which can also be combined with pedigree notes on a panel of morpho-phenological traits for breeding F1 hybrid varieties in the Radicchio of Chioggia biotype.

## Results

PCR-based amplifications of the genomic DNA samples from all inbred lines were performed to assay 27 mapped loci (information on primer pairs are reported in Additional file 1).

Descriptive statistics over all the SSR loci, along with information on the genetic diversity found across the molecular markers and plant accessions, are reported in Tables 1 and 2, respectively. The mean number of observed marker alleles (n_a_) in the SSR loci assayed was 5.9, varying from 3.3 in LG5 and LG7 to 9.3 in LG2 (Table 1). The frequency of the most common marker allele (p_i_) proved to be low when the observed number of marker alleles was high and vice versa (for instance, in LG2 and LG5, where the average p_i_ was 0.386 and 0.700, respectively). At the same time, both the expected heterozygosity (He) and Shannon’s information index of phenotypic diversity (I) were estimated to be high when the observed number of marker alleles was high (for instance, in LG2, where the average p_i_ was 0.749 and 1.610, respectively) (for additional statistics, see Table 1).

**Table 1:** Descriptive statistics of the SSR marker loci

		General statistics				H-statistics			F-statistics			Gene flow
Locus	N	p_i_	I	n_a_	n_e_	H_o_	H_e_	H_a_	F_is_	F_it_	F_st_	Nm
M1.1	400	0.423	0.741	3.0	2.009	0.165	0.503	0.117	0.017	0.827	0.824	0.054
M1.2	482	0.407	1.497	7.0	3.612	0.315	0.725	0.247	−0.055	0.640	0.658	0.130
M1.3	480	0.467	1.374	8.0	3.007	0.342	0.669	0.297	−0.154	0.471	0.542	0.212
LG1 mean	454	0.432	1.204	6.0	2.876	0.274	0.632	0.220	−0.064	0.646	0.675	0.132
M2.4	482	0.317	1.667	9.0	4.703	0.291	0.789	0.263	−0.249	0.588	0.670	0.123
M2.5	484	0.401	1.370	8.0	3.357	0.252	0.704	0.250	−0.086	0.610	0.641	0.140
M2.6	486	0.438	1.792	11.0	4.061	0.251	0.755	0.244	−0.134	0.634	0.677	0.119
LG2 mean	484	0.386	1.610	9.3	4.040	0.265	0.749	0.252	−0.156	0.611	0.663	0.127
M3.7	480	0.592	1.096	7.0	2.335	0.213	0.573	0.202	−0.194	0.590	0.657	0.131
M3.8	302	0.669	0.880	4.0	1.994	0.007	0.500	0.003	−0.067	0.995	0.996	0.001
M3.9	478	0.523	1.220	5.0	2.746	0.239	0.637	0.213	−0.320	0.580	0.682	0.117
LG3 mean	420	0.595	1.065	5.3	2.358	0.153	0.570	0.139	−0.193	0.722	0.778	0.083
M4.10b	486	0.410	1.225	4.0	3.074	0.165	0.676	0.194	0.014	0.720	0.716	0.099
M4.11b	482	0.847	0.481	3.0	1.360	0.017	0.265	0.052	0.632	0.927	0.801	0.062
M4.12	480	0.506	1.661	10.0	3.357	0.208	0.704	0.191	−0.103	0.699	0.727	0.094
LG4 mean	483	0.587	1.122	5.7	2.597	0.130	0.548	0.146	0.181	0.782	0.748	0.085
M5.13	476	0.517	1.129	4.0	2.684	0.202	0.629	0.184	−0.141	0.673	0.714	0.100
M5.14	474	0.884	0.359	2.0	1.258	0.114	0.206	0.102	−0.264	0.383	0.511	0.239
M5.15	478	0.699	0.754	4.0	1.796	0.092	0.444	0.074	−0.282	0.783	0.831	0.051
LG5 mean	476	0.700	0.748	3.3	1.913	0.136	0.426	0.120	−0.229	0.613	0.685	0.130
M6.16	476	0.546	1.304	5.0	2.835	0.181	0.649	0.177	−0.145	0.685	0.725	0.095
M6.17	480	0.469	1.707	9.0	3.750	0.358	0.735	0.293	−0.269	0.500	0.606	0.163
M6.18	480	0.527	1.495	8.0	3.063	0.208	0.675	0.169	−0.183	0.703	0.749	0.084
LG6 mean	479	0.514	1.502	7.3	3.216	0.249	0.686	0.213	−0.199	0.629	0.693	0.114
M7.19	480	0.554	0.996	3.0	2.460	0.479	0.595	0.346	−0.622	0.052	0.416	0.352
M7.20	484	0.531	0.739	4.0	2.023	0.236	0.507	0.241	−0.305	0.402	0.542	0.211
M7.21	474	0.878	0.390	3.0	1.275	0.076	0.216	0.099	−0.165	0.590	0.648	0.136
LG7 mean	479	0.654	0.708	3.3	1.919	0.264	0.439	0.228	−0.364	0.348	0.535	0.233
M8.22	480	0.990	0.058	2.0	1.021	0.021	0.021	0.016	−0.069	−0.009	0.056	4.192
M8.23	482	0.512	1.501	9.0	3.143	0.261	0.683	0.210	−0.307	0.605	0.698	0.108
M8.24	478	0.398	1.664	8.0	4.144	0.243	0.760	0.195	−0.245	0.675	0.739	0.088
LG8 mean	480	0.633	1.074	6.3	2.769	0.175	0.488	0.140	−0.207	0.424	0.498	1.463
M9.25	484	0.686	0.763	4.0	1.828	0.132	0.454	0.138	0.178	0.732	0.674	0.121
M9.26	478	0.490	1.354	6.0	3.101	0.088	0.679	0.176	0.459	0.860	0.741	0.087
M9.27	482	0.386	1.792	10.0	4.611	0.440	0.785	0.341	−0.327	0.425	0.567	0.191
LG9 mean	481	0.520	1.303	6.7	3.180	0.220	0.639	0.218	0.103	0.672	0.660	0.133

The sample size of individual genotypes (N), the frequency of the most common marker allele (p_i_), estimates of Shannon’s information index of phenotypic diversity (I), the average number of observed alleles (n_a_) and the effective number of alleles (n_e_) per locus, the observed heterozygosity (Ho), the expected heterozygosity computed using Levene (He), the average heterozygosity (Ha), Wright’s inbreeding coefficients F_is_ and F_it_, the fixation index (F_st_), and gene flow (N_m_)

**Table 2:** Descriptive statistics over all the accessions

	General statistics						H-statistics			F-statistics	
Line	nPl	%	I	SM	n_a_	n_e_	H_o_	H_e_	H_a_	F_is_	F_st_
P04	11	40.74	0.240	0.968	1.407	1.301	0.144	0.177	0.186	0.189	0.693
P31	18	66.67	0.429	0.925	1.741	1.573	0.291	0.313	0.186	0.070	0.457
P33	16	59.26	0.379	0.927	1.615	1.472	0.260	0.280	0.193	0.073	0.513
P11	11	40.74	0.279	0.950	1.423	1.379	0.234	0.212	0.193	−0.102	0.632
S02	8	29.63	0.210	0.976	1.346	1.281	0.130	0.156	0.189	0.167	0.729
S03	23	85.19	0.404	0.952	2.111	1.382	0.218	0.255	0.186	0.144	0.557
S31IS3.2	13	48.15	0.298	0.972	1.519	1.371	0.196	0.222	0.186	0.115	0.615
S31IS3.4	10	37.04	0.240	0.930	1.370	1.313	0.183	0.181	0.186	−0.013	0.686
S31S5.2	19	70.37	0.449	0.926	1.778	1.588	0.328	0.320	0.186	−0.024	0.444
S31S3	12	44.44	0.286	0.957	1.444	1.377	0.194	0.216	0.186	0.098	0.625
S31S5.1	20	74.07	0.477	0.961	2.000	1.585	0.284	0.351	0.186	0.190	0.391
Z22	10	37.04	0.202	0.966	1.407	1.229	0.130	0.142	0.186	0.087	0.753
Z23	16	59.26	0.389	0.949	1.741	1.503	0.301	0.278	0.186	−0.084	0.518
Z31	10	37.04	0.253	0.957	1.407	1.344	0.174	0.187	0.186	0.072	0.674
Z33	15	55.56	0.174	0.984	1.630	1.131	0.111	0.101	0.186	−0.104	0.825
Z34	7	25.93	0.160	0.989	1.259	1.215	0.111	0.120	0.186	0.073	0.792
U22	9	33.33	0.202	0.979	1.346	1.256	0.135	0.150	0.189	0.101	0.740
U24	13	48.15	0.304	0.945	1.519	1.409	0.199	0.224	0.186	0.112	0.611
QC03	18	66.67	0.455	0.941	1.692	1.608	0.307	0.344	0.193	0.108	0.402
QC31	7	25.93	0.199	0.971	1.385	1.274	0.164	0.136	0.193	−0.203	0.764
CS441	12	44.44	0.287	0.949	1.444	1.379	0.207	0.216	0.186	0.043	0.624
CS501	6	22.22	0.148	0.992	1.231	1.194	0.139	0.111	0.189	−0.260	0.808
SC24	5	18.52	0.129	0.971	1.192	1.177	0.125	0.098	0.193	−0.274	0.830
SE802	21	77.78	0.693	0.924	2.704	1.966	0.403	0.433	0.186	0.070	0.247
SE902	16	59.26	0.326	0.943	1.852	1.356	0.194	0.208	0.186	0.067	0.638
SE111S6	11	40.74	0.226	0.965	1.423	1.276	0.115	0.164	0.193	0.297	0.715
SEG111	5	18.52	0.139	1.000	1.200	1.200	0.200	0.200	0.194	0.000	0.652
SE111S7	8	29.63	0.222	0.990	1.423	1.295	0.202	0.155	0.193	−0.302	0.730
SE412	17	62.96	0.403	0.928	1.630	1.540	0.285	0.303	0.186	0.060	0.473
SE501	9	33.33	0.216	0.974	1.333	1.285	0.102	0.162	0.186	0.372	0.718
13	12	44.44	0.320	1.000	1.462	1.462	0.462	0.308	0.189	−0.500	0.465
11	8	29.63	0.213	1.000	1.308	1.308	0.308	0.205	0.189	−0.500	0.644
17	6	22.22	0.160	1.000	1.231	1.231	0.231	0.154	0.189	−0.501	0.733
49	11	40.74	0.293	1.000	1.423	1.423	0.423	0.282	0.189	−0.500	0.510
20	5	18.52	0.124	1.000	1.185	1.170	0.167	0.117	0.186	−0.421	0.796
38	6	22.22	0.149	1.000	1.222	1.207	0.204	0.142	0.186	−0.435	0.753
86	11	40.74	0.282	1.000	1.407	1.407	0.407	0.272	0.186	−0.500	0.528

The number of polymorphic loci (nPl), their frequency presented as percentage (%) of polymorphic loci on a total of 27 assayed, estimates of Shannon’s information index of phenotypic diversity (I), the genetic similarity coefficient or simple matching coefficient (SM), the average number of observed alleles (n_a_) and the effective number of alleles (n_e_), the observed heterozygosity (Ho), the expected heterozygosity computed using Levene (He), the average heterozygosity (Ha), Wright’s inbreeding coefficients F_is_ and F_it_ and the fixation index (F_st_)

The observed homozygosity scores were high, as expected for inbred lines, with a mean estimate of 0.793 (st. dev. = 0.120), and ranged from 0.521 to 0.993. The marker loci M3.8, M4.11b and M8.22, with observed heterozygosity values of 0.007, 0.017 and 0.021 (*i.e.*, homozygosity rates of 0.993, 0.983, 0.979), respectively, greatly contributed to this average homozygosity. Wright’s inbreeding coefficients (F-statistics) for single marker loci were also computed (Table 1). The inbreeding coefficient calculated for individual accessions revealed a negative value, on average equal to F_is_ = −0.125, as shown in Table 1. This feature was shared by 22 of the 27 SSR marker loci investigated, and it was particularly evident for the marker locus M7.19, which scored a very low observed homozygosity of 0.521. Values of the Wright’s fixation index, which were computed for each locus across LGs, are reported in Table 1. The average value was F_st_ = 0.659.

Estimates of gene flow (N_m_) were also computed for each locus (Table 1). The calculated values were slightly N_m_ > 0 for the vast majority of the assayed marker loci, ranging from a minimum of 0.001 to a maximum of 0.352, with an average value equal to N_m_ = 0.278.

Regarding the descriptive statistics over all the accessions, the number of polymorphic loci among individuals within inbred lines varied from 5 (18.5 %) to 23 (85.2 %) out of the total of 27 marker loci (Table 2). Simple matching coefficients scored values greater than 0.900 in all the accessions and were equal to 1.000 in eight. The observed homozygosity was on average high, with values greater than 0.800 in the majority of the inbred lines (ranging between 0.539 and 0.898). Conversely, the expected heterozygosity was on average law, varying from 0.098 to 0.433 (Table 2).

Principal coordinate analysis allowed for the definition of centroids for all the lines. The first two principal components explained 30.37 % of the total genetic variation found within the analyzed lines. Specifically, the first and second components explained more than 18 % and about 12 % of the total genetic diversity, respectively.

**Fig. 1:**
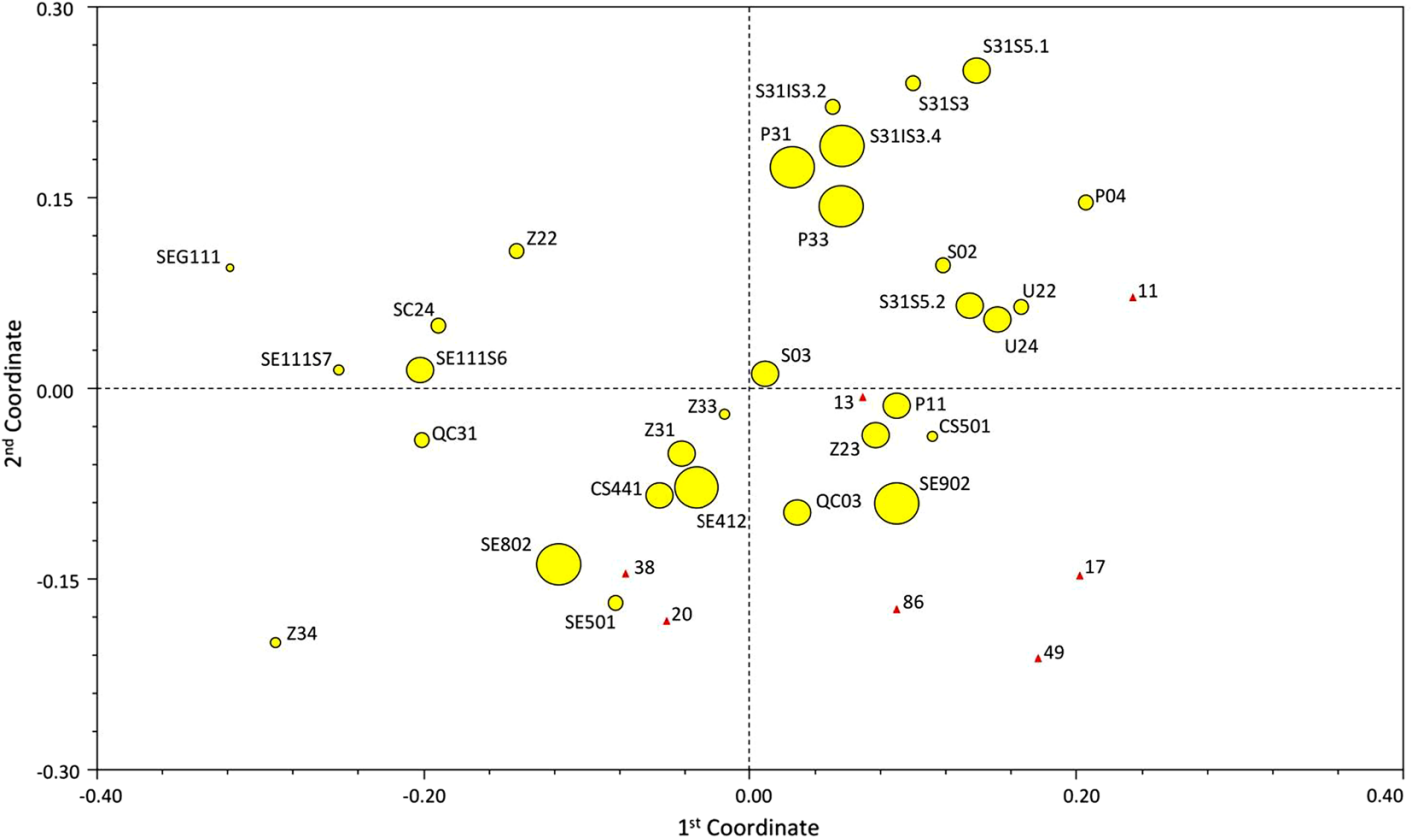
The centroids of all the inbred lines expressed as MGS (mean genetic similarity) estimates plotted according to the first two main components. The *red triangles* refer to the seed parents, whereas the *yellow dots* indicate the pollen donors. The difference in size is related to the genetic variability found within each accession represented by the standard deviation of the simple matching (SM) coefficient

Each inbred line, if regarded as a single centroid determined according to the mean genetic similarity (MGS) estimates, can be discriminated from the others on the basis of genotyping data, as shown in Fig. 1. A clear sub-grouping of inbred lines as single centroids was obtained in the four main quadrants when the individuals belonging to each accession were plotted bidimentionally according to the principal coordinates.

**Fig. 2:**
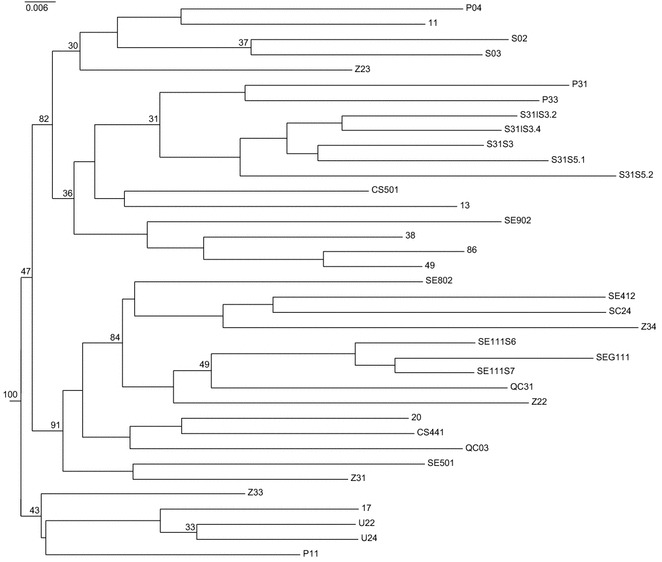
The NJ (neighbor joining) tree of the 37 inbred lines analyzed. The tree was computed using genetic dissimilarity matrix of all pair-wise comparisons between inbred lines. The *numbers* next to the main nodes indicate the bootstrap values (only estimates ≥30 % are reported)

A neighbor-joining (NJ) tree was also constructed on the basis of the genetic dissimilarity matrix whose mean coefficients were computed between all possible pair-wise combinations of inbred lines of the core collection (Fig. 2). Two main subgroups of branches with most of the accessions were generated each including about half of the inbred lines. Moreover, one of these sub-groups was further split into additional sub-nodes and two well-defined clusters wherein several inbred lines could be ordered. A few inbred lines were positioned apart from the main tree (Fig. 2).

The population structure was investigated using the ΔK method [11], which enabled to discover two levels of genetic grouping for the inbred lines (Additional file 2). When the number of accession units (K) was set to three (ΔK = 25), as many as 22 (59 %) of the inbred lines assayed in this study were grouped in a single main cluster, while two additional small clusters were formed each represented by six (16 %) inbred lines. Only four (11 %) genotypes displayed an admixed ancestry (*i*.*e*., membership <70 %), as expected with the occurrence of genetic recombination and hybridization.

**Fig. 3:**
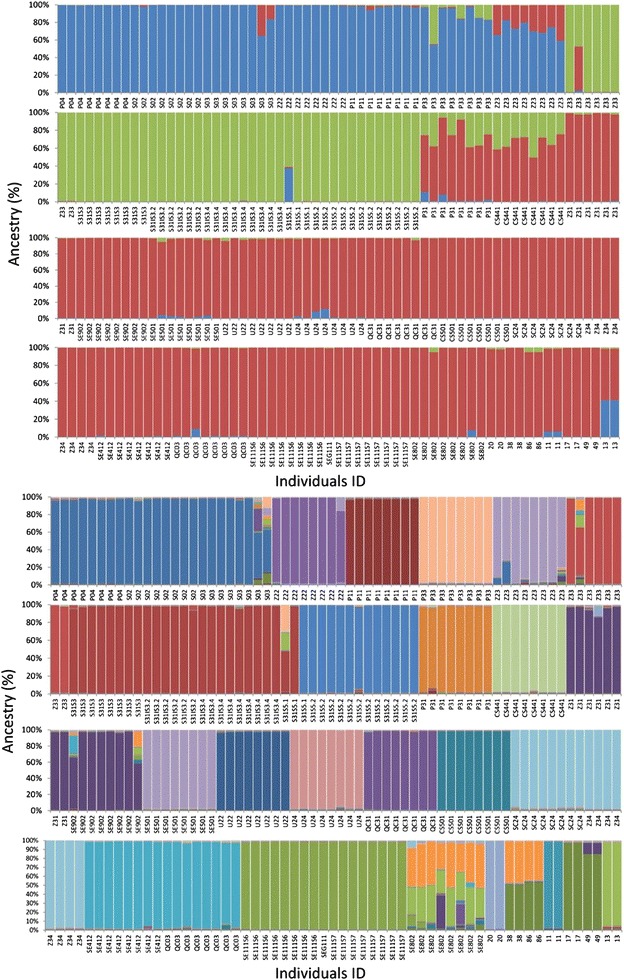
Estimated genetic clustering (K = 3 upper panel, and K = 24 lower panel) obtained with STRUCTURE. The population of inbred lines is reported on the *X axis*, whereas the percentage of ancestry is shown on the *Y axis*

A second level of genetic structure was investigated within groups of inbred lines (Additional file 2) by setting the number of accession units (K) to 24 (ΔK = 19). In this case, the whole collection of inbred lines was fragmented in clusters composed by one or few accessions (Fig. 3). In particular, the inbred lines S02, S03 and P04 shared high membership values to a single cluster, as well as the inbred lines SE111S6, SE111S7, SEG1111, and Z33, S31S3, S31S3.2, S31S3.4 were divided in two well-defined clusters. Four additional clusters were formed by couple of inbred lines sharing high membership to the same group. Overall, the proportion of individual genotypes that displayed admixed ancestry with membership to multiple clusters was equal to 8 %.

## Discussion

In this study, we developed a method for genotyping elite breeding stocks of leaf chicory (*Cichorium intybus* L.) by assaying microsatellite marker loci selected for the linkage map position and polymorphism information content. The genetic identity and stability of 37 inbred lines and the extent of their genetic diversity, expressed as genetic distance and allelic divergence, were addressed by using a panel of neutral SSR markers. Furthermore, our subset of 27 mapped SSR marker loci (*i*.*e*., 3 for each of the 9 basic LGs) proved to be very informative for the study of the population structure and inbreeding level of *C. intybus* plant materials. Along with estimates of observed homozygosity, the genetic stability of each breeding stock was addressed by computing Shannon’s estimates of phenotypic diversity and Rohlf’s coefficients of genetic similarity. On the whole, these statistics indicated strong genetic uniformity for the investigated inbred lines, as expected for breeding stocks developed by selfing and full-sibling programs. Furthermore, a marked inbreeding for the majority of the accessions was supported by the high homozygosity observed in nearly all the inbred lines (the mean value was as high as 78.7 %). Also, the Wright’s fixation index indicated that the genetic differentiation between the inbred lines is high (approximately 63 %) and that one-third of the genetic variation (approximately 33 %) is occurring within the inbred lines, due not only to homozygosity for different marker alleles but also for heterozygosity at some of the marker loci. Our data demonstrate that most of the genetic differentiation is occurring among inbred lines; thus, each breeding stock can be considered as genetically uniform and distinguished from the others of the core collection.

It is worth mentioning that individual inbreeding coefficients, as estimate of the strength of inbreeding for single inbred lines, were shown to be low or negative, indicating that the observed heterozygosity was greater than expected. Maintenance of such levels of heterozygosity in spite of inbreeding reproductive strategies (*i.e.*, selfing, full-sibling and back-crossing) could be a consequence of the reproductive system of *C. intybus,* which is naturally characterized by high frequency of allogamy as a result of self-incompatibility. We may also speculate that a fraction of the observed heterozygosity could be a consequence of phenotypic selection (*i.e.*, morphologically superior individuals) operated by breeders during inbreeding programs.

All mapped SSR markers exploited in this multi-locus DNA genotyping method scored high polymorphism information content, with the exception of marker M8.22 that revealed an almost monomorphic condition. Although its very low or null discriminant ability, this marker locus was taken into account as it showed an allele-specific genotype, which is typical of leaf chicory and allows to identify Radicchio from other *C.intybus* types (*e.g*., Witloof).

Regarding the NJ clustering results, the inbred lines known to be genetically related (*i.e.*, inbred lines that originated from the same local variety) proved to form a very well-defined subgroups of the tree (*e.g.*, accessions SE111 and S31). The STRUCTURE analysis of the population of genotypes (for K = 25) revealed clusters of single individuals in agreement with the grouping of inbred lines shown by the NJ tree analysis. In fact, inbred lines P04, S02 and S03 were grouped in the same cluster of ancestry and associated to the same node of the tree. This is also true for several pollen donor lines, such as SEG111, SE111S6 and SE111S7, and Z34 and SC24. In addition, the inbred lines belonging to the minor clade of the NJ tree were all grouped in different STRUCTURE clusters (see Figs. 2, 3 for details). As far as the seed parent lines (13, 20, 11, 38, 86, 17 and 49), they were grouped into four different clusters as expected on the basis of their variety of origin. Interestingly, the inbred line 13, which resulted to have an admixed ancestry, originates from a specific introgression and backcross program.

PCA allowed for the definition of centroids for all the inbred lines. The first component was positively associated with cycle length, discriminating long cycle accessions from short cycle accessions, with only a very few exceptions per class. It is also worth mentioning that the centroids of inbred lines with a common origin could be plotted in different areas of the quadrants. This finding suggests that it is possible to develop and select genotypically different inbred lines (*i.e.*, homozygous of different alleles at the same loci) starting from individuals selected within a given local variety of leaf chicory, namely Radicchio of Chioggia.

## Conclusions

Our research deals with the implementation and validation of a multi-locus genotyping system in leaf chicory that may prove to be useful for the marker-assisted breeding of new varieties of Radicchio. In particular, the plant materials used in this study cover a core collection of Radicchio of Chioggia (*i.e*., “Red of Chioggia” biotype) experimental materials, which not only manifest valuable traits, but also possess applicable uses in modern breeding programs.

From a technical point of view, labeling each set of primers with different fluorescent dyes allowed us to differentiate and score up to eight SSR marker loci in a single Genescan^®^ run. One important advantage of this method is a substantial cost savings for fluorescent primer labeling, because the synthesis of a specific fluorescently labeled primer for each SSR marker locus is not needed. The multiplex-ready PCR required only four fluorescent-dye labeled primers to complete the research analyses. Furthermore, multiplex-ready PCR combines both the advantages of the M13-tailed primer method [12] and multiplex PCR [13] for fluorescent-based SSR genotyping of single individuals. The use of the M13 primer has several advantages over other techniques. First, it allows for working with a unique tail sequence and avoiding the need of using the requirement of several different SSR dye labeled primers. In addition, the technique has the further advantage of being less time consuming and reducing consumable costs. For multiplex purposes, it is only necessary to change the fluorescent colors to label the different PCR products of each SSR marker locus. We were therefore able to reconstruct the genotype of each individual across all the accessions for as many as 27 target loci (3 selected marker loci for each of the 9 LGs) by performing 14 PCR reactions and 4 Genescan^®^ runs.

In conclusion, we successfully developed and implemented an efficient and reproducible method for the multi-locus genotyping of elite breeding stocks of leaf chicory belonging to the Radicchio of Chioggia biotype using 27 microsatellite marker loci scattered throughout the genome. We demonstrated that this method is useful for assessing the homozygosity and genetic stability of single inbred lines and for measuring the specific combining ability between maternal and paternal inbred lines on the basis of their genetic diversity. This information could be exploited for planning crosses and predicting the heterosis of experimental F1 hybrids on the basis of the allelic divergence and genetic distance of the parental lines. Knowing the parental genotypes would enable not only to protect newly registered varieties but also to assess the genetic purity and identity of the seed stocks of commercial F1 hybrids, and to certificate the origin of their food derivatives.

## Methods

### Plant materials and DNA isolation

Plant materials of the “Red of Chioggia” biotype, belonging to *C. intybus* subsp. intybus var. *foliosum* L. (Table 3), were developed and provided by T&T Produce (Sant’Anna di Chioggia, Venice, Italy). Most of the inbred lines were represented by pollen donors spanning from S3 to S7 obtained by repeated selfing of single individuals chosen within each progeny at both genotype and phenotype levels (see upper part of Table 3). Lines coded as IS3 were partial inbreds derived from intercrossing closely related S3 progeny plants. A few male-sterile seed parents were selected within F2 progenies obtained by selfing of F1 individuals, FS1 progenies produced by full-sibling and S1BC1 progenies generated by selfing of BC1 individuals (see lower part of Table 3). Concerning the strategy of sampling, the pollen parents were represented by eight individuals per accession, with few exceptions, whereas the seed parents were propagated by in vitro culture and represented each by replicated individuals of clonal lines. All plant materials were bred in an experimental station at Chioggia (Venice, Italy) under controlled pollination conditions.

**Table 3:** Plant materials

Accession ID	No. individuals	Population type	Varietal cycle (d)
P04	8	S4	55
P31	8	S4	55
P33	8	S4	55
P11	8	S5	55
S02	8	S4	65
S03	8	S4	65
S31IS3.2	6	IS3	65
S31IS3.4	9	IS3	65
S31S5.2	12	S5	65
S31S3	8	S3	65
S31S5.1	3	S5	65
Z22	8	S4	75
Z23	8	S4	75
Z31	8	S5	75
Z33	8	S4	75
Z34	8	S4	75
U22	8	S2	75
U24	8	S2	75
QC03	8	S5	90
QC31	8	S5	90
CS441	8	S6	110
CS501	8	S6	110
SC24	8	S5	120
SE802	8	S6	140
SE902	8	S6	140
SE111S6	8	S6	140
SEG111	1	S6	140
SE111S7	8	S7	140
SE412	8	S6	140
SE501	8	S6	140
13	1	S1BC1	65
11	1	F2	80
17	1	FS1	90
49	1	F2	90
20	1	F2	110
38	1	FS1	110
86	1	F2	110

Information on the plant materials, including the inbred line ID, the number of individuals assayed per population, the inbred level reached per each line and the cycle of the variety each line derives from, expressed in days after transplanting. The number of generations of selfing (S) is reported for pollen donors, whereas full-sibling (FS), back-crossing (BC), pair-wise crossing (F) between inbred lines or inter-crossing between selfed individuals (IS) refers to seed parents. In addition, male inbred lines were multiplied in vivo by seeds (*i*.*e*., inbreeding) and female inbred lines were propagated in vitro by cuttings (*i*.*e*., cloning)

The genomic DNA was extracted from 100 mg of fresh leaves with the GenElute™ Plant Genomic DNA Miniprep Kit (Sigma–Aldrich, http://www.sigmaaldrich.com) following the manufacturer’s instructions. The quality of the DNA samples was assessed by electrophoresis on 1 % (w/v) agarose gel stained with 1X SYBR^®^ Safe™ DNA Gel Stain (Life Technologies) in Tris–Acetate-EDTA (TAE) running buffer. The yield and purity of the extracted genomic DNA samples were evaluated using a NanoDrop 2000c UV–Vis Spectrophotometer (Thermo Scientific). Following DNA quantification, all the DNA samples were diluted to a final concentration of 25 ng/μl to be used as template for PCR amplifications.

### Amplification of SSR loci

**Fig. 4:**
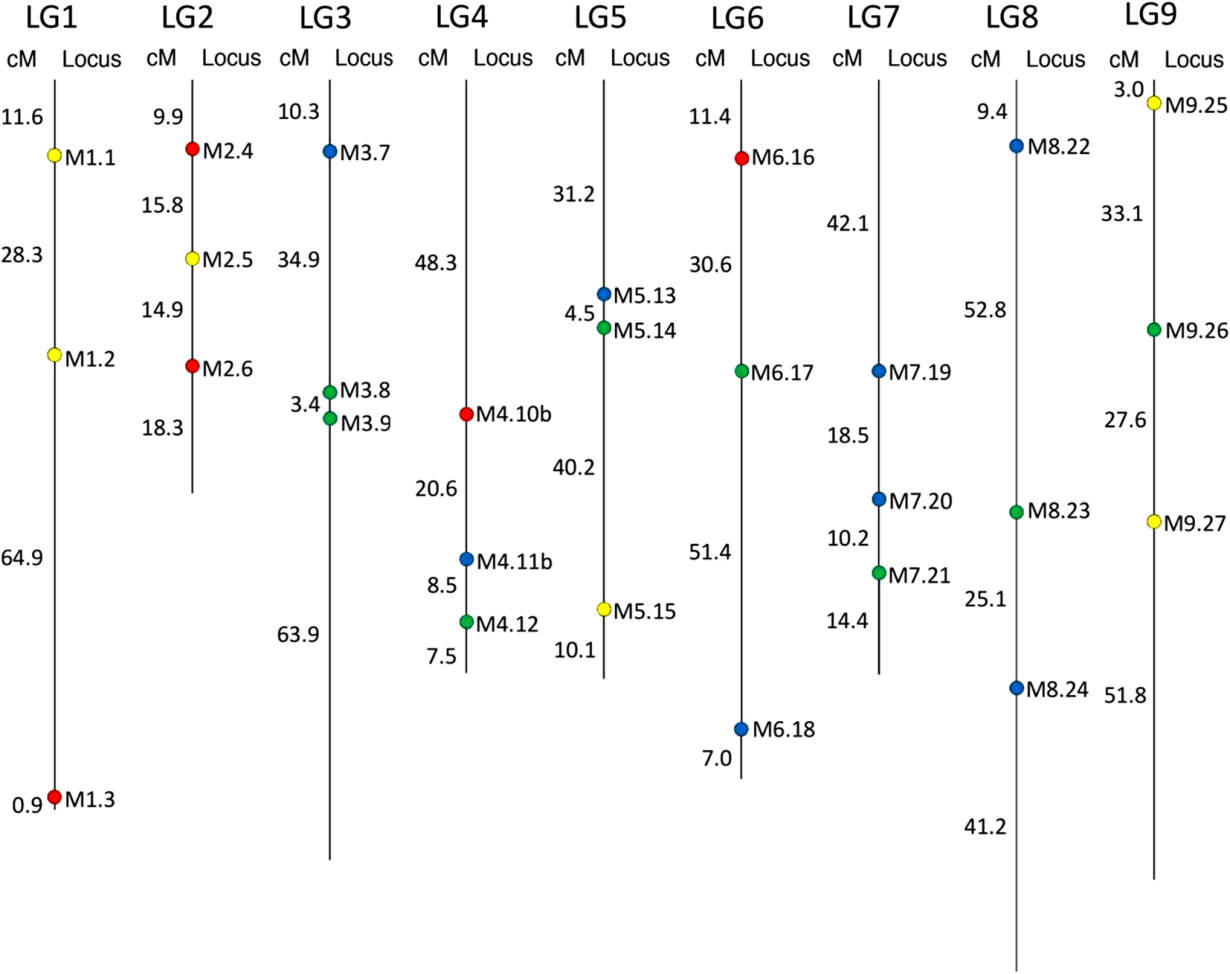
Consensus genetic linkage map of chicory (*C. intybus*), modified from Cadalen and coll. [10]. The *colored dots* indicate the positions of the selected marker loci throughout the nine basic LGs. Each color represents the fluorophore used to label microsatellite-containing amplicons related to each locus. The position of the marker loci and the length of each LG are also reported

A total of 27 SSR marker loci were selected among those mapped in the nine basic LGs constructed for *C. intybus* [10]. In particular, three SSR loci were carefully chosen for each LG in order to select the best ones in terms of polymorphism information content (PIC) scores and also to be well scattered throughout the genetic map (Fig. 4). The amplification of microsatellites was performed by using a PCR multiplex assay and the detection of DNA fragments across marker loci was achieved using a 5′ M13-tailed primer method [12] with some modifications. Only one dye-labeled M13 primer was used per PCR reaction in combination with any other M13-tailed forward primer [14]. The SSR motifs and primers used in this study are described in Additional file 1.

Amplification reactions were set in order to analyze two marker loci with the same fluorescent dye in each PCR experiment [14]. In general, the marker alleles produced for different target loci, assayed with the same fluorescent dye labels, were characterized by distinct amplicons deriving from PCRs of similar efficiency, as DNA markers were combined in order to obtain reproducible amplicons of different size ranges.

The reactions were performed in a total volume of 20 µl including 2X Platinum^®^ Multiplex PCR Master Mix, 10X GC Enhancer (Applied Biosystems, Carlsbad CA), 0.25 µM of each tailed primer, 0.75 µM of each non-tailed primer, 0.5 µM of each labeled primer (Invitrogen, Carlsbad CA), 25 ng of DNA and distilled water. The amplification reactions were performed in a Gene Amp^®^ PCR System 9700 thermal cycler (Applied Biosystems).

The microsatellite-containing regions were amplified using two different PCR cycles defined to maximize the amplification efficiency of the different primer sets according to their annealing temperatures, as reported in Additional file 1, as well as the fluorescent dye labels and the two-loci matching system to perform the multi-locus PCR reactions.

The cycle termed 'rad-multi 54' consisted of an amplification reaction performed under the following conditions: 95 °C for 5 min followed by 5 cycles at 95 °C for 30 s, 58 °C for 45 s decreasing by 0.8 °C every cycle, 68 °C for 45 s followed by 35 cycles at 95 °C for 30 s, 54 °C for 45 s, 68 °C for 45 s and a final extension of 60 min at 68 °C. Similarly, the cycle 'rad-multi 56' consisted of the following conditions: 95 °C for 5 min followed by 5 cycles at 95 °C for 30 s, 60 °C for 45 s decreasing by 0.8 °C every cycle, 68 °C for 45 s followed by 35 cycles at 95 °C for 30 s, 56 °C for 45 s, 68 °C for 45 s and a final extension of 60 min at 68 °C.

The quality of the PCR products was assessed by electrophoresis on a 2 % (w/v) Agarose gel stained with 1X SYBR^®^ Safe™ DNA Gel Stain (Life Technologies) using Tris–Acetate-EDTA (TAE) running buffer. The concentrations of the PCR products were estimated with KODAK 1D Image Analysis Software by comparing the intensity of the PCR products to that of a 1 Kb Plus DNA Ladder (Life Technologies). Then, for each PCR reaction and fluorophore, approximately 20 ng of amplification products were pooled and prepared for capillary electrophoresis.

### Analysis of the SSR loci

DNA fragment capillary electrophoresis was completed at BMR Genomics (Padova, Italy). Following electrophoresis, fragment analyses were performed with Peak Scanner^Tm^ v. 1.0 (Life Technologies).

Owing to the relatively recent origin of the biotype “Red of Chioggia” (*i*.*e*., years 1960–1970s), belonging to the same botanical variety (*i*.*e*., *Cichorium intybus* subsp. *intybus* var. *foliosum* L.), we assumed the absence of homoplasy and adopted an infinite alleles model, hence considering that marker alleles of the same size at a given locus had the same evolutionary history. Statistical analyses of the SSRs were performed with the PopGene software package v. 1.32 [15] for calculating allele frequencies: the alleles per locus (n_e_), Levene’s [16] observed heterozygosity (Ho), the expected heterozygosity (He) and the average heterozygosity (Ha) were computed for each locus per line. PopGene, software was also used to estimate F-statistics [17]. Estimates of the heterozygosity within (F_is_) and between (F_it_) subpopulations were determined, as was the fixation index (F_st_) according to Wright [18].

The phenotypic diversity of the marker allele profiles was estimated using Shannon’s information index (I) as reported by Lewontin [19]. Gene flow (N_m_) estimates among the subpopulations were derived from the fixation index as described by McDermott and McDonald [20].

The NJ dendrogram of all the accessions was based on Nei’s method [21]. A bootstrap analysis was conducted with 1000 resampling replicates. The Dice’s coefficient [22] was applied to calculate the proportion of genetic similarity (GS) in all the pair-wise comparisons of individuals. Values of the genetic similarity calculated with Rohlf’s coefficient were used to conduct PCA, and the results are represented as centroids plotted according to the MGS estimates.

All the relevant calculations and analyses were conducted using the appropriate routines in the NTSYS software package v. 2.21c [23].

The population structure of the inbred red Chioggia chicory lines was investigated using the model-based (Bayesian) clustering algorithm implemented in the STRUCTURE software [24], which groups individuals according to marker allele combination and distribution. All the simulations were executed assuming an admixture model with no a priori population information. The calculations were performed with 300,000 iterations and 300,000 burn-ins under the assumption that the allele frequencies in the populations were correlated. Fifteen replicate runs were performed, with each run exploring a range spanning 1–35 K. The most likely value of K was estimated using ΔK, as reported in other studies [11]. The individuals with membership coefficients of qi > 0.8 were assigned to specific groups, whereas the individuals with qi < 0.8 were characterized identified as being admixed.

## Additional files

10.1186/s13104-015-1819-z List of primer pairs used to amplify the SSR containing regions.
10.1186/s13104-015-1819-z Output of the ΔK method [11] showing the two investigated levels of K (*i.e.* K = 3 and K = 24).

## Abbreviations

AFLP: amplified fragment length polymorphism
LG: linkage group
MAB: marker assisted breeding
MAS: marker assisted selection
MGS: mean genetic similarity
PCA: principal component analysis
PCR: polymerase chain reaction
RAPD: random amplified polymorphic DNA
SSR: Simple Sequence Repeat
st. dev.: standard deviation
TAE: tris acetate EDTA
NJ: Neighbor Joining
USA: United States of America
