# Supplementary material for: A method for genotyping elite breeding stocks of leaf chicory (Cichorium intybus L.) by assaying mapped microsatellite marker loci
Source: BMC Res Notes. 2015 Dec 30;8:831. doi: 10.1186/s13104-015-1819-z (PMC4696096; doi:10.1186/s13104-015-1819-z)
Supplement: Supplementary file 1 — 10.1186/s13104-015-1819-z List of primer pairs used to amplify the SSR containing regions. [file 13104_2015_1819_MOESM1_ESM.pdf]

Additional File 1. List of primer pairs used to amplify the SSR containing regions

List of primer pairs used to amplify the SSR containing regions, along with information on the individual SSR motifs and LG to which each SRR marker locus belongs. The M13 tailed primers are indicated in bold. The dotted lines separate the multilocus PCRs; the plain lines indicate Genescan® runs.

Old ID[1]	New ID[2]	LG	Motif		Primer sequence	Tm (°C)	GC (%)	PCR cycle	Dye
**EU03D01**	M7.19	7	$(CT)_{18}$	For	ATGTCGGAGCAAAATCGTTC	59.38	45	Rad-multi 56	6-FAM
				Rev	**TTGTAAAACGACGGCCAGT**CATGTTCCCGCTCATGAATA	58.21	45		
**EU10H05**	M7.20	7	$(CT)_{31}$	For	**TTGTAAAACGACGGCCAGT**GTCATGATGGCGTAAAAG	53.95	44	Rad-multi 56	
				Rev	ACACTCACTCACACTCCGTAA	61.25	47		
**EU02C09**	M4.12	4	$(CT)_8TT(CT)_5CC$ $(CT)_3TT(CT)_7$	For	GGCATCGGGATAGAAAAACA	58.43	45	Rad-multi 56	VIC
				Rev	**TTGTAAAACGACGGCCAGT**TCAATGCCTCAACAGAAATCC	59.04	42		
**EU07G10**	M4.10b	4	$(CT)_{21}CATA$ $(CA)_5\,CT(CA)_5$	For	CATCCATTATTGGGCAG	52.33	47	Rad-multi 54	PET
				Rev	**TGTAAAACGACGGCCAGT**CACCAACGAACTCCTTACAAA	58.92	42		
**EU0022**	M1.1	1	$(GA)_{40}$	For	**TTGTAAAACGACGGCCAGT**CCAACGGATACCAAGGTGTT	60.45	50	Rad-multi 54	NED
				Rev	AACCGCACGGGTTCTATG	60.18	55		
**A124**	M9.25	9	$(CA)_{11}$	For	GTGTGGGTGTTTGAAGAGC	59.43	52	Rad-multi 54	
				Rev	**TTGTAAAACGACGGCCAGT**TCAAGAACATCAACGCGTAA	58.31	40		
**EU03H01**	M4.11b	4	$(TG)_5CG(TG)_7$	For	GCCATTCCTTTCAAGAGCAG	59.66	50	Rad-multi 56	6-FAM
				Rev	**TTGTAAAACGACGGCCAGT**AACCCAAAACCGCAACAATA	59.07	40		
**EU01H08**	M3.7	3	$(CT)_{22}$	For	TTCGAGTCTTGCCTTAATTGTT	58.75	36	Rad-multi 56	
				Rev	**TTGTAAAACGACGGCCAGT**CAGACGACCTTACGGCAACT	62.64	55		
**A149**	M3.9	3	$(CA)_{12}$	For	**TTGTAAAACGACGGCCAGT**CTGCTATGGACAGTTCCAGT	59.58	50	Rad-multi 56	VIC
				Rev	CAATTCAGTTGTGATAGACGC	57.81	42		
**EU11C09**	M3.8	3	$(CT)_{16}$	For	AGGAAGCGGTGTCATCTGT	61.40	52	Rad-multi 56	
				Rev	**TTGTAAAACGACGGCCAGT**CGCCCACATATTCATTCTCA	58.21	45		
**sw2H09.2**	M2.5	2	$(CT)_5CC(CT)_{13}$ $TT\,(CT)_5$	For	GTGCCGGTCTTCAGGTTACA	62.60	55	Rad-multi 54	NED
				Rev	**TTGTAAAACGACGGCCAGT**CGCCTACCGATTACGATTGA	60.16	50		
**sw2A12.2**	M9.27	9	$(GA)_{10}TAAA$ $(GA)_5$	For	**TTGTAAAACGACGGCCAGT**GCTAAAAGAAGTGCAAGGAGA	58.70	42	Rad-multi 54	
				Rev	TGTTCTTTCAAGTGCCAA	54.94	38		
**B42**	M2.6	2	$(CT)_{26}$	For	**TTGTAAAACGACGGCCAGT**GGAGCAGGTAGAGTCCCATC	61.61	60	Rad-multi 54	PET
				Rev	CGTTTGAAAATTTATACCAAAATG	54.54	25		
**EU07F12**	M6.16	6	$(CT)_{12}TT(CT)_{15}$ $TT\,(CT)_2TT(CT)_4$	For	**TTGTAAAACGACGGCCAGT**TATTGCATTGTTGTTCCTTG	54.90	35	Rad-multi 54	
				Rev	TGTATTTAGAAGAGGGAAATAGATG	56.44	32		

Old ID[1]	New ID[2]	LG	Motif		Primer sequence	Tm (°C)	GC (%)	PCR cycle	Dye
**EU07B09**	M8.22	8	$(CA)_5AA(CA)_9$	For	TCGTCATCAGAAACAAAGCAA	58.92	38	Rad-multi 56	6-FAM
				Rev	**TTGTAAAACGACGGCCAGT**CAAAGAAGGCACTCTTGTCG	59.60	50		
**A94**	M8.24	8	$(TC)_{16}(CA)_{13}$	For	**TTGTAAAACGACGGCCAGT**GGTCCGTAGACTGCAGACTTT	62.27	52	Rad-multi 56	
				Rev	CACCGTCCCACTTTTTAGG	58.52	52		
**B214**	M5.14	5	$(TC)_{11}$	For	**TTGTAAAACGACGGCCAGT**AAAGTCACACATCGCATTTCCT	61.32	40	Rad-multi 56	VIC
				Rev	GTAGCAGCAGCAGCCATCTT	63.38	55		
**sw2F09**	M9.26	9	$(GATA)_3N_{19}(GA)_9$	For	**TTGTAAAACGACGGCCAGT**CCTACACTCGGCCACCTACT	63.36	60	Rad-multi 56	
				Rev	TCGACGGTATAACAACACCTG	60.28	47		
**EU02E02**	M7.21	1	$(CT)_{13}$	For	GGACACCGAGCTGGAGAA	61.46	61	Rad-multi 56	
				Rev	**TTGTAAAACGACGGCCAGT**TTCCACTTTCGGGAGTTACC	59.80	50		
**EU06C09**	M1.3	1	$(CT)_{17}$	For	**TTGTAAAACGACGGCCAGT**TGGAGAAAAATGAAGCAC	53.16	38	Rad-multi 54	PET
				Rev	GAATGAGTGAGAGAATGATAGGG	58.36	43		
**B131**	M2.4	2	$(GA)_{25}$	For	**TTGTAAAACGACGGCCAGT**GCTCGAAAATCGGCTACAAC	60.09	57	Rad-multi 54	
				Rev	CGAGCCATGTTAGGGTTTGT	60.81	50		
**EU08C07**	M6.18	6	$(CT)_{16}$	For	CTCAACGAATGCTTTGGACA	59.23	45	Rad-multi 56	6-FAM
				Rev	**TTGTAAAACGACGGCCAGT**CCTCGCGGTAGCTTATTGTT	60.66	50		
**EU02A11**	M5.13	5	$(CT)_{23}$	For	AGGCATAAAGAGGTGTGG	56.61	50	Rad-multi 56	
				Rev	**TTGTAAAACGACGGCCAGT**TCAAACATGAAAACCGCTC	56.89	42		
**EU07C10**	M8.23	8	$(CA)_{11}(CT)_9$	For	TGTAGACACACAAAATGCACA	58.84	38	Rad-multi 54	VIC
				Rev	**TTGTAAAACGACGGCCAGT**ACCGGTTGAAAACATGAAAT	56.59	35		
**A158**	M6.17	6	$(CA)_8(CT)_{18}$	For	**TGTAAAACGACGGCCAGT**CGTGTCCAAACGCAAACATTAT	60.85	40	Rad-multi 54	
				Rev	GCACAATTTTCCTACCACTTATCC	60.42	41		
**EU0030**	M5.15	5	$(CT)_{11}N_7(CAA)_5$	For	**TTGTAAAACGACGGCCAGT**AGCACGACTCTGCTGTCTTTT	62.75	47	Rad-multi 54	NED
				Rev	CGAGCCATGTTAGGGTTTGT	60.81	50		
**EU02D02**	M1.2	7	$(CT)_{19}$	For	**TTGTAAAACGACGGCCAGT**CCGGCAGAATTTTTAGGG	56.14	50	Rad-multi 54	
				Rev	CAGGTCATAGGTCCATGTGAAA	60.27	45		

1. Cadalen *et. al.* [10]

2. Ghedina *et. al.* [present paper]
