# Supplementary figures and images for: A method for genotyping elite breeding stocks of leaf chicory (Cichorium intybus L.) by assaying mapped microsatellite marker loci
Source: BMC Res Notes. 2015 Dec 30;8:831. doi: 10.1186/s13104-015-1819-z (PMC4696096; doi:10.1186/s13104-015-1819-z)

$\Delta K$  Radicchio - 27 loci SSR

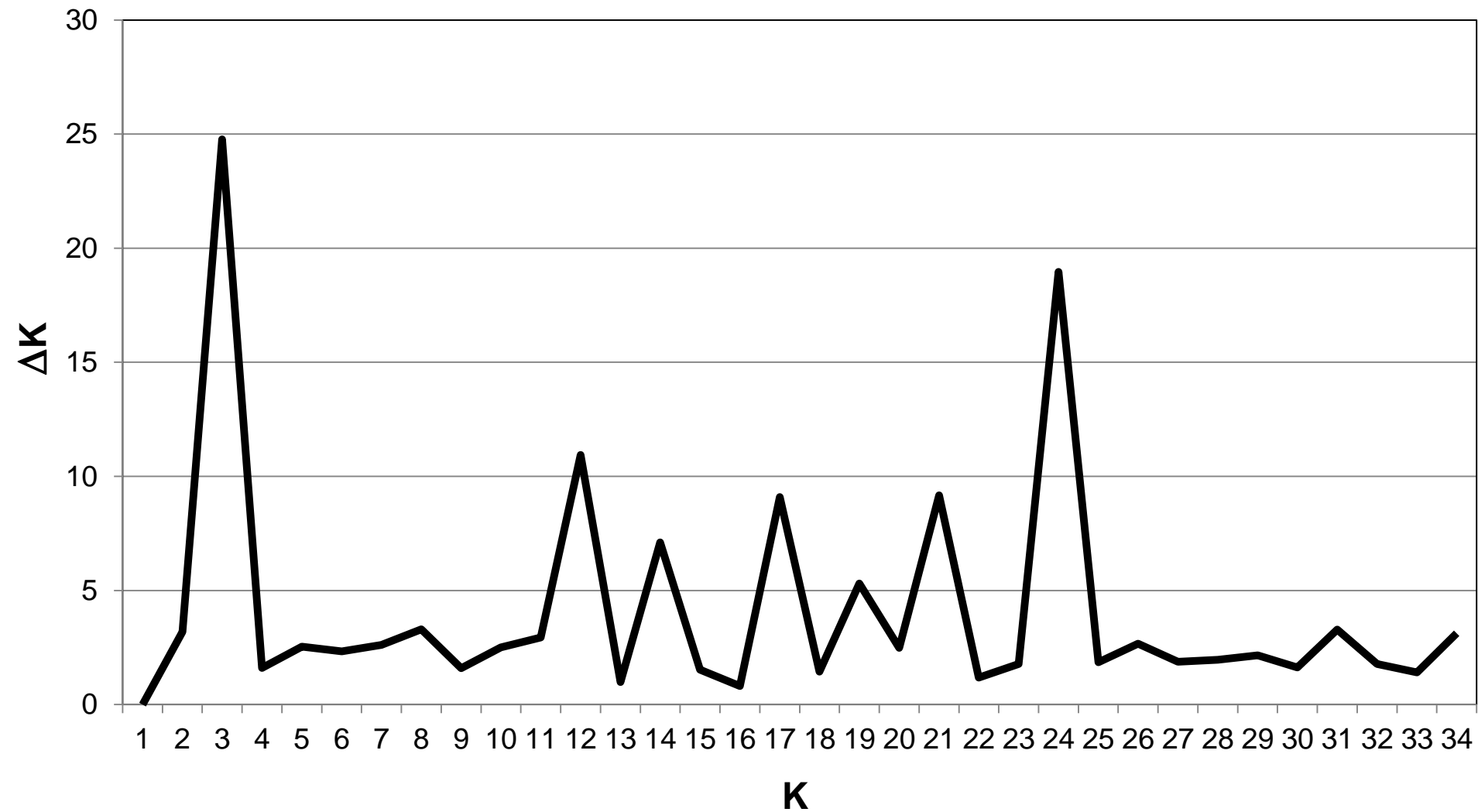

Supplement: Supplementary file 2 — 10.1186/s13104-015-1819-z Output of the ΔK method [11] showing the two investigated levels of K (i.e. K = 3 and K = 24). [file 13104_2015_1819_MOESM2_ESM.pdf]
